# Challenges and Opportunities of Implementing Data Fusion in Process Analytical Technology—A Review

**DOI:** 10.3390/molecules27154846

**Published:** 2022-07-28

**Authors:** Tibor Casian, Brigitta Nagy, Béla Kovács, Dorián László Galata, Edit Hirsch, Attila Farkas

**Affiliations:** 1 Department of Pharmaceutical Technology and Biopharmacy, “Iuliu Hatieganu” University of Medicine and Pharmacy, 400012 Cluj-Napoca, Romania; casian.tibor@umfcluj.ro; 2 Department of Organic Chemistry and Technology, Budapest University of Technology and Economics, H-1111 Budapest, Hungary; galata.dorian.laszlo@vbk.bme.hu (D.L.G.); hirsch.edit@vbk.bme.hu (E.H.); farkas.attila@vbk.bme.hu (A.F.); 3 Department of Biochemistry and Environmental Chemistry, George Emil Palade University of Medicine, Pharmacy, Science, and Technology of Târgu Mureș, 540139 Târgu Mureș, Romania; bela.kovacs@umfst.ro

**Keywords:** data fusion, process analytical technology, chemometrics, process control

## Abstract

The release of the FDA’s guidance on Process Analytical Technology has motivated and supported the pharmaceutical industry to deliver consistent quality medicine by acquiring a deeper understanding of the product performance and process interplay. The technical opportunities to reach this high-level control have considerably evolved since 2004 due to the development of advanced analytical sensors and chemometric tools. However, their transfer to the highly regulated pharmaceutical sector has been limited. To this respect, data fusion strategies have been extensively applied in different sectors, such as food or chemical, to provide a more robust performance of the analytical platforms. This survey evaluates the challenges and opportunities of implementing data fusion within the PAT concept by identifying transfer opportunities from other sectors. Special attention is given to the data types available from pharmaceutical manufacturing and their compatibility with data fusion strategies. Furthermore, the integration into Pharma 4.0 is discussed.

## 1. Introduction

The pharmaceutical industry has witnessed substantial changes from a regulatory perspective in the past few decades, aiming to ensure the quality of the pharmaceutical product by a thorough understanding of both the product particularities and the manufacturing thereof [1]. The adoption of the ICH Q8-10 guidelines and the elaboration of the concept of design of experiments by pioneering researchers in this field represented notable milestones in the quality management of pharmaceutical products [2,3,4]. Concurrently to these, the appearance of the Food and Drug Administration’s (FDA) guidance on Process Analytical Technology (PAT) in 2004 forecasted an important paradigm shift of the major regulatory bodies according to which quality cannot be tested in products; it should be built-in or should be by design [5].

The driving force of many pharmaceutical companies to introduce PAT in their manufacturing environment is referring to the reduced batch failures and reprocessing, production process optimization, and faster release testing with the opportunity to enable real-time release testing through feedback and feedforward control loops [6]. The immediate financial benefit/impact of a PAT-based control strategy translates into an increase in production yield and a reduction in manufacturing costs. The increased amount of data obtained from monitoring can further guide the optimization and continuous improvement of the system, generating additional monetary value [7]. This ability to monitor a process in real-time and obtain an improved understanding of product-process interplay requires appropriate tools (PAT instruments) that can track the right product attributes [6].

Process monitoring can be performed with various instruments, from built-in univariate sensors to more complex sensors that can be interfaced with the process stream. Both options could be very efficient if sufficient data is used to design these process control tools to support their use. Thus, the reliability of a PAT procedure for the manufacturing requirements and the selected control strategy is conditioned by its design, performance qualification, and ongoing performance verification within proper lifecycle management [8].

The major challenges associated with the adoption of PAT in the pharmaceutical industry refer to the integration of the probe, the sampling interface, data collection, modeling, linking to a control system, the calibration of the method, and finally, the validation of the integral system. Frequently, these high throughput instruments produce large datasets recorded over multiple variables, requiring specialized data analysis methods. In this respect, the European Directorate for the Quality of Medicines and Healthcare issued the “Chemometric methods applied to analytical data” monograph in 2016 to encourage using these analysis methods as an integral part of PAT applications [8].

**Figure 1:**
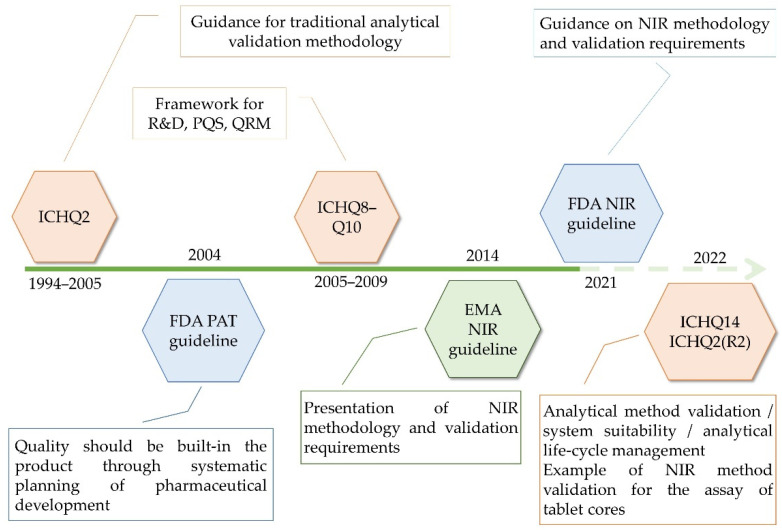
Guidelines used for the quality management of pharmaceutical products.

As demands for the application of advanced technologies have increased, regulatory documents aimed to formulate specific frameworks regarding the analytical development and validation methodologies to facilitate the application of chemometrics in pharma. As such, guidelines by the European Medicines Agency (EMA) and FDA have been elaborated, dealing with the development and data requirements for submitting Near Infrared Spectroscopy (NIR) procedures in 2014 and 2021, respectively. Meanwhile, new ICH guidelines have been considered—ICHQ13 and ICHQ14—having in sight the principles of continuous manufacturing technology and the analytical quality by design (QbD) approach [4]. Furthermore, with the elaboration of ICHQ14, the ICHQ2 guideline is currently under review, with both concept papers being endorsed for public consultations on the 24 March 2022 (Figure 1).

PAT is an indispensable unit in the newly emerging continuous manufacturing technologies and is required to demonstrate the process state of control and detect quality variations. Continuously recorded data enables the detection of process deviations and supports the root cause analysis of such events and the opportunity for continuous improvement [8].

Drug products present a complex quality profile built around multiple critical quality attributes (CQAs) influenced by controlled (formulation and process) and uncontrolled factors. A multivariate approach to product/process understanding is critical due to the complex interactions between these input factors affecting product quality. Moreover, these factors are likely to have different influence patterns between several quality attributes. To efficiently describe and understand these influences, a Design of Experiments-based development with response surface methodology is recommended [3,4].

If the recorded data accounts for multiple factors influencing that particular response, predicting complex quality attributes from PAT data can be managed appropriately from only one data source. Under these circumstances, the variation of any influential factor will be captured/perceived in the process analytical data and contribute to the method’s robust predictive performance. Thus, to obtain a robust monitoring performance, it is essential to identify the PAT tool sensible to these factors or to fuse multiple process analytical data.

The readily available advanced analytical platforms provide large amounts of diverse data associated with manufacturing processes that can be used for monitoring and predictive purposes. The challenge, in this case, refers to the integration of data from different sources to maximize the advantages of complementary information. The underlying idea/notion in performing data fusion (DF) is that the result of the fused dataset will be more informative than the individual datasets. Thus, this procedure will provide a more enhanced overview of the studied system with a more in-depth understanding and data-driven decision-making [9,10,11].

Implementing the DF concept in PAT represents the next step in the evolution of process monitoring technology that could provide a more comprehensive understanding of the system and the opportunity to predict complex quality attributes of drug products. Probably, due to the more strictly regulated field of the pharmaceutical industry, the use of this concept in drug manufacturing has been limited to some extent.

Several review papers are available on DF, focusing on the chemometric/data processing or the application side of data integration. Azcarate et al. published a review on DF, focusing on the structure of data originating from different sources along with DF strategies [12]. Mishra et al. reviewed the application of multi-block analysis methods for multi-source data integration, highlighting the advantages, disadvantages, and particularities of different techniques [13]. On the same subject, Campos et al. reviewed the pre-processing methods for multiblock applications [14]. Moreover, a relevant review on the application of pre-processing strategies and pre-processing fusion approaches is available from Mishra et al. [15].

On the application side of DF, food applications predominate. Zhou et al. reviewed the application of DF technology in food quality authentication applications, providing an effective comparison with non-fusion approaches [16]. Borras et al. provided a general overview of DF strategies implemented for food and beverage characterization [17]. Two other reviews are available on the application of artificial senses in food quality assessment [18,19].

This review evaluates the challenges and opportunities of implementing DF in the pharmaceutical industry, namely PAT, considering applications from other sectors. The manuscript is organized into five sections. The first part focuses on the pharmaceutical domain’s data types, considering small molecule processing and biotechnology. The second part presents the concept behind DF, data processing, and modeling strategies. The third section reviews the use of DF in classification, regression, and process control applications, focusing on the interplay between input data structure, DF strategy, and performance improvement. Moreover, attention is given to the handling of spectroscopic data. The fourth part discusses the validation of these models, detailing the methodology used to evaluate the performance of these models in the surveyed literature and the expectations from a regulatory point of view. The last part presents the integration into Pharma 4.0 and some future perspectives.

The overall purpose of this work is to provide a systematic summary of all the key elements that must be considered during the use of DF within PAT applications and to support its implementation in real-life situations.

## 2. Data Types in the Pharmaceutical Industry

### 2.1. Off-Line Acquired Material Information

The utility of data acquired during routine in-process control (IPC) measurements can be extended if used as input in models that predict the behavior of processes where these manufactured materials are used. These measurements can characterize the composition of the samples (most commonly active pharmaceutical ingredient—API content, moisture/residual solvent content, and the concentration of contaminants). Another relevant type of information comes from the granulometric characterization of powders. This includes the particle size distribution (PSD) (typically measured with laser diffraction or sieve analysis), the shape of particles (characterized by static or dynamic image analysis), the density of the powder (bulk, tapped, or true density), and the flowability (flowing time, angle of repose, Carr index, Hausner ratio). Tablet cores can be evaluated by measuring their mass, diameter, height, crushing strength, disintegration time, and friability. Furthermore, all techniques mentioned in the next part can also be applied as off-line tools for IPC measurements [20,21].

### 2.2. Real-Time Measured Data

**Figure 2:**
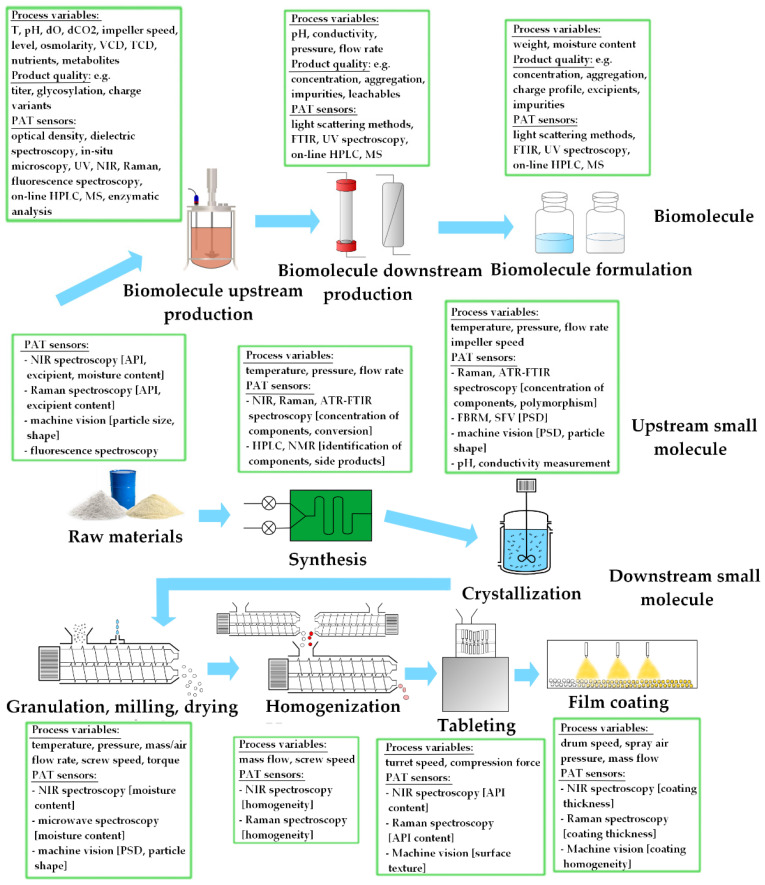
Data types encountered in the pharmaceutical industry.

Nowadays, a great variety of real-time sensors is available in pharmaceutical manufacturing (Figure 2) [22,23,24]. The dimensionality of the yielded data varies significantly from simple numbers to large three-dimensional matrices. In this respect, we can differentiate between zero-, first- and second-order structures. Zeroth-order data contains one response per sample, first-order data describes sample properties using multiple variables (a vector), whereas second-order data includes a matrix for each sample [12].

One-dimensional (zeroth-order) data is acquired when measuring some of the fundamental physical properties of the system. Temperature is a classic example; it is a critical parameter in many processes, including chemical reactions, granulation, and film coating. Its real-time measurement can be accomplished with various instruments. Thermocouples are a widespread solution, as they can be installed in multiple places inside an appliance [25]. The measurement of pressure is essential in many instances, as apart from influencing the quality of the product, its monitoring is a fundamental part of preventing accidents. The amount of applied force is a crucial parameter of compaction processes. Thus, it should be registered during dry granulation and tableting. The accurate real-time measurement of weight with scales is vital in continuous manufacturing, where the mass flow of the components is determined by the feeding rate of the feeders [26]. Moreover, real-time weight measurement is also used in batch processes to keep track of the amount of dosed material during wet granulation or film coating.

Monitoring the applied torque during high-shear or continuous twin-screw wet granulation can be used to characterize the state of the process, as the fill level of the apparatus and the granular properties of the processed material can influence this parameter [27,28]. The rotational speed of impellers in chemical or crystallization reactors and granulation appliances and the speed of the drum in film coating can also be registered [29]. The volume flow and moisture content of air can also be critical parameters in the case of fluidized bed granulation, drying, or film coating. The pH and conductivity value of the medium can be measured with in-line electrode probes during chemical reactions of crystallization [30].

Many analytical sensors provide two-dimensional (first-order) data, such as spectroscopic information and particle size distribution data. In-line measurement of the particle size distribution can be realized with probes based on various principles. Spatial Filter Velocimetry (SFV) [31] and Focused Beam Reflectance Measurement (FBRM) [32] characterize the chord length of the particles, while methods based on digital imaging such as Particle Vision Measurement (PVM) [33], or Eyecon^®^ give information about the two-or three-dimensional shape of the particles [34]. The data obtained from these sensors usually consists of the volume fraction of particles of different sizes.

Due to the dynamic evolution of spectroscopic techniques, most forms of spectroscopy can now be performed in-line or on-line with commercially available instruments. Their signal consists of the absorbance or intensity measured at multiple wavelengths. Typically, this information needs to be processed using multivariate data analysis techniques before being used as input in a DF process model. Near-infrared (NIR) [35] and Raman spectroscopy [36] can be applied in almost all types of pharmaceutical processes, as they can be used to predict the composition and various physical properties of intermediate and end products. Microwave sensors have been proposed as an alternative for quantifying the composition of pharmaceutical products [37]. The concentration of some APIs can be monitored using the light-induced fluorescence method [38]. Attenuated total reflectance Fourier transform infrared (ATR-FTIR) spectroscopy can also be used in situ in liquid phase systems, having widespread applications in monitoring crystallization [39]. In this field, attenuated total reflection ultraviolet/visible (ATR-UV/Vis) spectroscopy can also be utilized to measure the concentration of components [40]. Terahertz spectroscopy has also become an option for characterizing solid-state pharmaceutical products [41]. With an appropriate sampling system, even nuclear magnetic resonance (NMR) spectroscopy and high-performance liquid chromatography (HPLC) measurements can be performed on-line, providing an unparalleled ability to understand and control chemical syntheses [42]. Furthermore, even the sound emitted by an apparatus can be used to gain information about its state. Acoustic emission measurements are designed for this purpose [43]. In summary, the great flexibility of spectroscopy makes these techniques excellent PAT sensors.

The most complex form of information comes from imaging appliances. The recorded signal enables the characterization of sample features’ spatial distribution. Digital images are the simplest example of such techniques; machine vision is a sensor that can be applied in-line during practically all pharmaceutical processes [34]. It is a highly flexible tool that can characterize samples’ size, shape, texture, and color. Optical coherence tomography is an imaging technique with promising abilities in the real-time monitoring of film coating, as the obtained images enable an accurate measurement of coating thickness [44]. Terahertz pulsed imaging can also be applied for this purpose [45]. Hyperspectral imaging records a spectrum at each pixel of the image, enabling the prediction of the samples’ composition in each pixel. Raman [46], UV, and NIR spectroscopy can all be used to obtain hyperspectral images, UV [47] and NIR imaging [48] already exist in applicable real-time forms.

### 2.3. Biopharmaceutical Aspects

The manufacturing of most biopharmaceuticals (except DNA/RNA and peptides) includes production using bioreactor cell cultivation, chromatographic purifications, filtration steps, and formulation either in a liquid or solid form. Several methods are used to monitor CQAs of raw materials and critical process parameters (CPPs) as real-time data during these processes.

The cell culture media’s quality is of utmost importance to maintain process robustness. It usually contains various substances (>50) in a relatively low concentration. Thus, to characterize media quality, NMR, HPLC-MS/MS, and spectroscopic methods, such as fluorescence- (2D, 3D), infrared- (NIR, MIR, FT-IR), or Raman spectroscopy are used, resulting in complex multi-dimensional data [49,50,51,52,53,54]. In addition, the advantages of multivariate data analysis and DF methods can be utilized to gain accurate information on media quality [55].

Real-time measurements of basic physicochemical parameters (such as temperature, pH, conductivity, dissolved O_2_ and CO_2_, impeller speed, pressure, flow rate, weight, and moisture content) resulting in one-dimensional data are conventionally carried out during biopharmaceutical production [56,57]. However, gaining information on the cells and monitoring nutrient and metabolite concentrations during bioreactor cell cultivation is also necessary [58]. Optical density sensors measure the transmitted light absorbance, which correlates to total cell density. However, it gives no information on viability. Dielectric spectroscopy can be used to determine viable cell density, where the capacitance of the cell suspension is measured in an alternating frequency electric field, generating multi-dimensional data [59]. If the morphology of the cells is an essential factor, in situ microscopy aided with image analysis can be implemented in the bioreactor [60].

Spectroscopic methods (UV-, NIR-, Raman- and Fluorescence spectroscopy) have applications for monitoring several cell culture parameters, such as nutrient and metabolite concentrations, total and viable cell density, product concentration, and product quality [61,62]. Raman spectroscopy is gaining importance in biopharmaceutical manufacturing as a multi-attribute multi-dimensional sensor due to its specificity and compatibility with aqueous solutions [63]. During the purification of the biomolecules, monitoring of product concentration and impurities is possible with spectroscopic methods [64]. Besides the conventionally used UV absorbance at 280 nm as one-dimensional data or as a multiwavelength method, variable pathlength UV spectroscopy allows the accurate detection of analytes in a high concentration range [65]. Furthermore, several analytical techniques are used to detect aggregates in a wide size range, from which only a few can be integrated as an in-line PAT tool (e.g., light scattering methods) [66,67]. When there is no available in-line analytical tool for monitoring a CQA/CPP, an automated, sterile sampling system can be integrated into the process. This is the case for several CQAs where the integration of an online sampling and sample preparation system coupled with HPLC or HPLC-MS can be applied [68].

## 3. Data Fusion

### 3.1. Classification and Comparison of Fusion Methods

**Figure 3:**
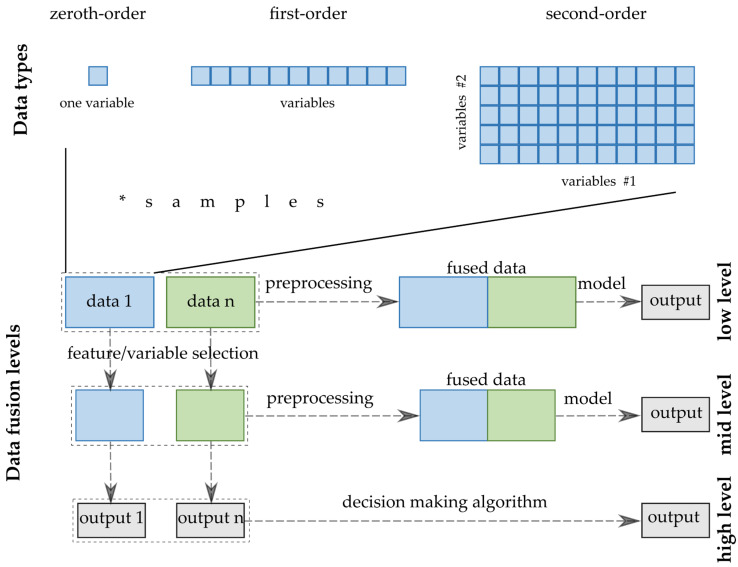
-DF strategies and data structures.

Several aspects exist that are used to classify the fusion methods/strategies in the terminology. Joint Directors of Laboratories (JDL) Data Fusion Group worked out a model that deals with the categorization of the information and DF. Castanedo systematized the classification of the DF techniques and strategies [69]. The divisions can be created by several criteria; however, the widespread classification used to accept in analytical chemistry follows the abstraction level of the input data [70]. The three levels are named after the complexity of the processing of the inputs from the data sources. Thus, low-, medium- and high-level DFs are distinguished (Figure 3).

Low-level data fusion (LLDF) is considered the simplest method to achieve a combination of inputs. In this case, the data is rearranged into a new data matrix, where the variables coming from different sources are placed one after the other. The columns, i.e., the variables of the combined data matrix, will be the sum of the previously separated data sets. Usually, the concatenated data are then pretreated before creating the final classification or regression models. However, specific elementary operations can be conducted before putting them together [17].

Medium-(mid-)level data fusion (MLDF) (also called “feature-level” fusion) is based on a preliminary feature extraction that continues to maintain the relevant variables, eliminating the not sufficiently diverse, non-informative variables from the datasets. There are many developed algorithms to select these features or make the data reduction before merging them into one matrix that will be used in a chemometric method [71]. In detail, these variable selection methods are discussed with the other preprocessing methods in Section 3.2.

The high-level data fusion (HLDF) (also called “decision-level” fusion) works on a decision level. This means that the first step is to fit some supervised models to each data matrix. These models consist of regression models providing continuous responses for the input data or classifications, deciding the class membership of the new samples. The decisions from these models are combined into a complex model that can create the final estimation. The main idea behind HLDF is that the optimal regressions and classifications are built up for the different data types. Accordingly, a better estimation may be reached by unifying the outputs in one decision model.

Selecting and implementing an appropriate fusion method can prove to be a laborious task and should be driven by the considered application and the structure of the input data. To provide an effective comparison of the method’s performance in different setups (application type/input data structure), a literature survey was performed using studies that compare different fusion levels (Appendix A Table A1). Considering the pharmaceutical industry, the main areas of application of DF would include classification, regression, and process control, whereas regarding the data structure, mainly zero- and first-order data are encountered. Thus, all these factors/criteria were considered in the survey.

**Figure 4:**
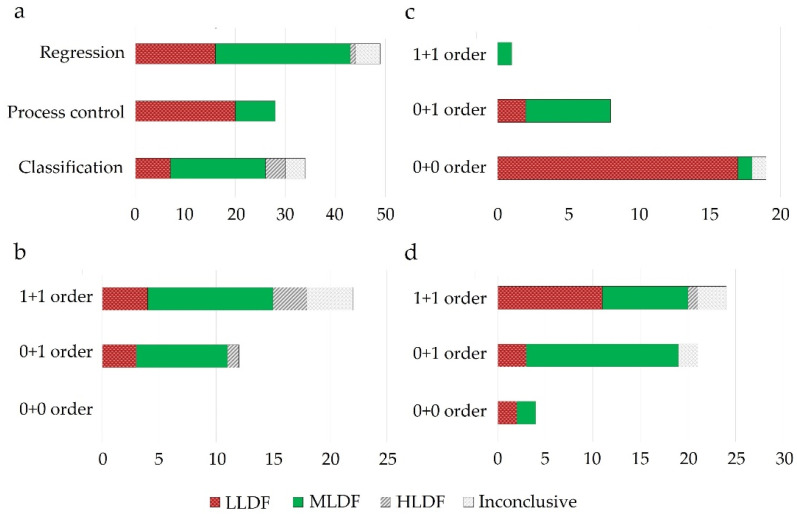
Evaluation of the best performing DF strategies across different areas of application (**a**) and their selection according to the data structure used for modeling ((**b**)-classification, (**c**)-process control, (**d**)-regression applications); 0 + 0: fusion of zeroth order data; 0 + 1: fusion of zeroth order data with first order data; 1 + 1: fusion of first-order data; *x*-axis represents the number of studies.

LLDF predominated as a suitable DF option under process control applications, where primarily multiple zero-order datasets were fused for multivariate- (MSPC) or batch statistical process control (BSPC) purposes (Figure 4). This strategy also proved effective for regression applications to merge several first-order datasets. Therefore, the fusion of data with a similar structure was efficient without applying a feature extraction procedure, as the similar structure avoided the predominance of one dataset over the other. Increased performance of LLDF was also attributed to the existence of complementary information between the datasets, which was maintained during the fusion procedure (not lost during feature extraction) [72]. Having more complementary information will be beneficial for reducing uncertainty.

In some situations, instrument complementarity (not data complementarity) was not sufficient to improve the performance of predictive several CQAs, as shown by a Raman and FT-IR data based food analytical study [73].

As LLDF involves the concatenation of individual blocks at the level of original matrices after proper preprocessing, the dataset will contain many variables, some with increased predictive power, and also large parts of irrelevant data [72]. The ratio of predictive and uninformative variables obtained by adding new data can be disadvantageous as the noise can cancel out the advantages of valuable information [11,74,75]. Thus, the model building can become time-consuming and requires high computational power, although this limitation was overcome by using extreme learning machine modeling with a fast learning speed [76].

Assis et al. found the LLDF superior to MLDF when fusing NIR with total reflection X-ray fluorescence spectrometry (TXRF) data, highlighting the importance of scaling and variable selection procedure on the fused dataset. Autoscaling outperformed the block-scaling approach, and a variable reduction procedure was essential to eliminate redundant information [77]. A similar method was found appropriate by Assis et al. when ATR-FTIR and paper-spray mass spectrometry (PS-MS) data were combined [78].

Li et al. also demonstrated the superiority of LLDF over MLDF when NIR and MIR data were fused. The partial loss of relevant information during feature extraction affected MLDF performance [79]. As both LLDF and HLDF approaches relied on using the full spectral range, the developed models were superior to MLDF [79]. In this respect, the disadvantage of MLDF refers to the requirement of thoroughly investigating various feature extraction methods by developing multiple individual models [72,74]. However, the time invested in this stage is compensated by the more efficient model development using the extracted features [80].

MLDF was preferred when first-order data was combined with a zero-order or another first-order dataset (Figure 4). MLDF outperformed other fusion strategies when the feature extraction methods successfully excluded the uncorrelated variables.

If the extraction of features does not lead to the loss of predictive information, the MLDF strategy can offer a more accurate model and improved stability [81]. Therefore, the desired outcome of feature extraction is to maximize the amount of predictive variable content and minimize data size [82].

MLDF can offer a more balanced representation of variability captured in each dataset, especially when the number of variables is considerably large. The increased stability and robustness of MLDF over LLDF were also described in other studies [75,83,84]. The high level of redundant information found in LLDF data, negatively affected the synergistic effect of the fusion for different datasets [75,82,85].

A huge amount of information is involved when handling spectroscopic data. Thus, feature extraction is frequently implemented. Perfect classification of sample origin was achieved by separately extracting features from three different spectroscopic analysis techniques (NIR, fluorescence spectroscopy, and laser-induced breakdown spectroscopy (LIBS)) [86]. A similar discrimination model with successful identification was demonstrated for tablets using LIBS and IR spectra and MDLF [87].

Among the three areas of application, HLDF was selected as the best performing mainly in the case of classification applications when fusing first-order datasets (Figure 3). The utility of HLDF was also highlighted under similar input conditions in the case of regression applications (Figure 4).

Li et al. demonstrated that the synergistic effect of fusing data (FT-MIR; NIR) was achieved only when the valuable part of the data was used. LLDF was poorly performing due to the increased content of useless data, whereas the best classification strategy relied on HLDF [82]. The application dependency for selecting the fusion strategy has been recognized in other studies [72]. Another NIR and MIR-based application demonstrated the superior performance of HLDF, as the LLDF led to the loss of complementary information in the large dataset. At the same time, the MLDF approach gave mixed results depending on the evaluated response [11]. The use of the entire dataset over extracted features was the reason for HLDF superiority in another study [79].

In a previous study, LLDF caused no progression in classification, as presumably the analytical methods and sensors had dissimilar efficiency and provided noisy and redundant data [88]. Therefore, each output of the models had to be considered with different weights to make the final decision.

The advantages of HLDF are linked to its user-friendliness [11], and the possibility to easily update models with new data sources increases the versatility [89].

### 3.2. Data Processing

Regardless of the specific goal of the DF, the data measured by the analytical tools and sensors must be processed by various methods before building up chemometric models.

Firstly, the data sets might have different sizes, scales, and magnitudes. This can be handled by normalization and standardization to rescale the values into a range or to zero mean and unit variance. Autoscaling could be an appropriate solution for the fusion of univariate sensors with multivariate data, which frequently occurs in chemical or pharmaceutical processes [90,91,92,93]. The min-max normalization is suitable for MS [94] and some vibrational spectroscopic data [86,95]. It is typical to use normalization methods or elemental peak ratios for LIBS data to minimize the variability of replicates [96].

In the absence of differences in the measurement scale, additional preprocessing methods (scaling methods) will not be necessary, as the chance of dominating behavior will be reduced. This situation was encountered when mid-wave infrared (MWIR) and low-wave infrared (LWIR) data recorded by the same device were fused [72].

Secondly, the data, especially the spectral data, is usually influenced by the external interferences and measuring conditions causing different backgrounds, noise, and offset. Many well-known methods exist to increase the robustness of the datasets and, later, the models. Savitzky–Golay smoothing (SGS) is a commonly used method for noise reduction in spectra [79,85,97]. Several methods are proposed to tackle additive and/or multiplicative effects in spectral data. Background correction (BC) [98], Multiplicative Scatter Correction (MSC) [99], and Standard Normal Variate (SNV) [82,100] Unit area and vector normalization [98] are possible transformation methods to compensate for these effects. First or second derivatives are beneficial for enhancing the slight changes, thus, separating peaks of overlapping bands [40,75,101].

Thirdly, a dimensionality reduction step is essential to extract relevant features in MLDF [91,102,103]. Another justification for this step is to reduce the computational time during model development, i.e., for neural networks [104,105].

**Figure 5:**
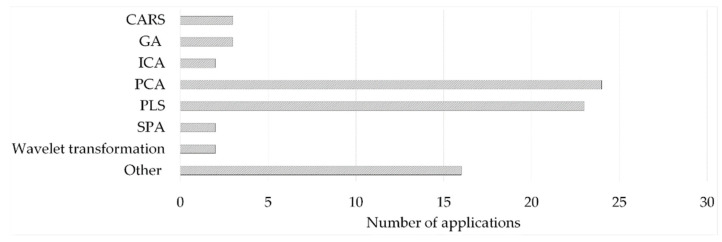
(Other—1 entry/method: 2D-image based estimator; correlation-based feature selection-CFS; forward selection; IRIV; multivariate curve resolution-alternating least squares (MCR-ALS); PARAFAC; Random frog (RF); Spectral signatures and leaf venation feature extraction; spectral window selection (SWS); T2, Q—derived from NIR-based MSPC; UV; variable selection based on the normalized differences between reference and sample spectral data; Variables Combination Population Analysis and Iterative Retained Information Variable Algorithm—VCPA-IRIV).

The applied feature extraction strategies identified in the literature survey can be divided into feature selection procedures relying on algorithms for selecting a sub-interval of the original dataset or on dimensionality reduction procedures, such as projection methods [76]. Moreover, their combined use has been demonstrated to have positive results in some situations [75,78,85]. The feature extraction methods applied in the literature survey for first-order data are presented in Figure 5.

The measured data, particularly the spectral data, often include irrelevant variables that should be separated from the initial variables. Variable selection algorithms eliminate noisy spectral regions and redundant information to increase predictive accuracy [75]. In this respect, several methods derived from partial least squares (PLS) have been used. The synergy interval PLS (SI-PLS) algorithm was applied to select optimal subintervals and exclude unwanted sources of variation before a feature extraction step [75,85]. De Oliviera et al. reduced the variable numbers from LIBS and NIR spectra below 1% by recursive PLS (rPLS) and used them for DF purposes [106].

Uninformative and noise affected variables have been excluded using interval-PLS (i-PLS) [107,108]. As i-PLS continuously selects the variables, it should not be applied when the original data are not continuous (i.e., MS spectra) [78]. The use of variable importance in the projection (VIP) and i-PLS has also been reported [100].

The VIP-based variable ranking has shown efficacy in filtering unimportant variables and reducing variable space [84,108,109]. Generally, a VIP > 1 is considered relevant, although this limit has no statistical meaning [84,99,110]. In this respect, Rivera-Perez et al. identified discriminant variables through VIP and an additional statistical significance criterium (*p* < 0.05) from ANOVA or *t*-tests [111].

The use of genetic algorithm (GA), iteratively retained informative variables (IRIV), competitive adaptive reweighted sampling (CARS), successive projections algorithm (SPA), recursive feature extraction (RFE), univariate filter (UF), and ordered predictors selection (OPS) has also been reported [78,86,94,108,110,112,113]. GAs have been used in spectroscopic applications for optimal wavelength selection, multicollinearity, and noise reduction [108]. The algorithm selects an initial set of spectral variables, which is further optimized by testing multiple combinations of different features. The comparison between GA and UF [113], respectively, and GA and OPS variable selection methods has been investigated for DF applications [78].

The fine-tuning of variables can be dealt with individually for each data set at the statistical significance level, through Pearson correlation analysis [114]. Another option that enables the extraction of features from spectroscopic data is wavelet transformation. During this procedure, the original signal is decomposed considering different wavelet scales, resulting in a series of coefficients [115]. Wavelet compression was used for the fusion of spectral data from different sources [107], while other studies fused different scale-based wavelet coefficients generated from the same input data [115].

The other big category of feature extraction methods relies on estimating a new set of variables. Projection methods were the most frequently applied feature extraction tools to reduce the dimensionality and remove unwanted correlation. More than 60% of the studies included in this survey used either Principal component analysis (PCA) or PLS for this purpose during the development of fusion-based models. Both techniques are based on the coordinate transformation of the original n × λ sized dataset (where n is the number of observations and λ is the number of variables) by combining the original variables. In the case of PCA, this is performed in the way that the new variables (i.e., principal components, PCs) are orthogonal, and the first few variables describe the possible highest variance in the dataset. For PLS, the new variables (latent variables, LVs) maximize the covariance with the dependent variables. For more details, the reader is referred to, e.g., [116] and [117]. Other feature extraction methods found in the literature are parallel factor analysis (PARAFAC), a generalization of PCA [91], independent component analysis (ICA) [118], orthogonal-PLS [104], or autoencoder [119].

The obtained LVs have been extensively used as relevant features for DF applications [118,120,121,122]; for overview purposes [104] and outlier identification [72].

The use of latent variables as extracted features has to consider the size of captured variability [96,104,105]. In this respect, several significance criteria have been used for selecting relevant PCs, including the percentage of explained original data (R2X) [82,123], the eigenvalue [104] or the predictive performance during cross-validation (RMSECV) [124,125]. Some applications excluded the possibility of discarding relevant PCs and fused multiple latent variables, independent of their significance [76,126,127]. However, such an approach increases the risk of overfitting.

The use of the Gerchberg–Saxton algorithm has also been reported to establish the optimal number of feature components [75].

Several studies found PLS to be a superior feature extraction method, as it was possible to emphasize the spectral variability correlated with the response of interest [125,128]. For example, Lan et al. extracted the features of interest from NIR spectra by developing PLS models having as a response the components of interest determined by HPLC [110].

The separation of spectral variability into predictive and orthogonal parts can be achieved using orthogonal-PLS (OPLS). As a result, the feature extraction can efficiently exclude uncorrelated variations from the input data [104]. Although it would appear beneficial to use only the predictive components, non-predictive parts can have a positive effect on performance results due to the intra-class correlations from different sources [129].

PLS-DA (PLS-Discriminant analysis), another extension of PLS, has also been applied for feature extraction [74], by either generating latent variables [11] or by selecting a small set of representative variables [80].

### 3.3. Modeling Methods

**Figure 6:**
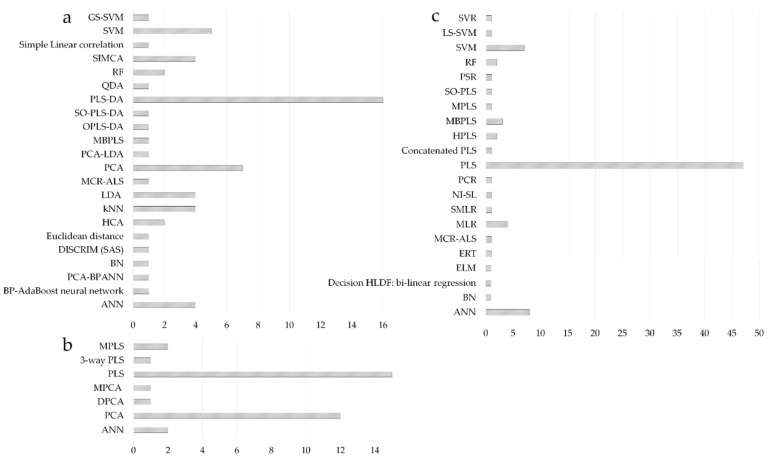
Evaluation of modeling methods considered for classification (**a**); process control (**b**) and regression purposes (**c**); *x*-axis represents the number of studies using a particular method.

PCA and PLS regression can be regarded as the most widespread chemometric tools [130]; consequently, the literature survey highlighted the predominance of projection methods in the modeling of fused datasets (Figure 6). PLS-DA and PCA were the preferred modeling choice for classification applications, followed by the support vector machine (SVM), soft independent modeling using class analogy (SIMCA), linear discriminant analysis (LDA), k-nearest neighbors (kNN), or artificial neural networks (ANN) (Figure 6a). For process control applications, PLS models were mainly used to develop batch evolution/level models having process maturity (time-variable) or a CQA of the product as response variables (Figure 6b). PLS was also the preferred modeling option for regression applications, followed by ANN and SVM methods (Figure 6c).

In the case of LLDF, PCA and PLS modeling can be directly applied to analyze the different data sources. This is an especially suitable method when univariate sensor data are fused, such as in [131], as the computational demand of the model might increase significantly when several multivariate data (e.g., spectra with thousands of variables) are handled together. Nevertheless, it is also possible to concatenate different spectra for developing a single PCA or PLS model. For example, mid- and long-wave NIR spectra could be incorporated into the same PLS model to utilize the information of the whole IR range [72]. The only difference between PCA/PLS models developed for DF—compared to a single-source model—is that additional preprocessing steps (see Section 3.2.) might be necessary to compensate for the possible scale differences.

Several extensions of the traditional PCA/PLS concept account for the structured nature of the fused dataset. For instance, Multiblock-PLS (MB-PLS) provides block scores, as well as relative importance measures for the individual data blocks instead of accounting for the whole concatenated data [132]. Although the prediction itself does not improve compared to the traditional PLS model, it significantly contributes to the interpretability of the model. For example, the block weights and scores have helped identify the most critical variables in an API fermentation [133]. In other studies, MB-PLS and the “block importance in prediction (BIP)” index were used to determine which PAT sensors (IR, Raman, laser-induced fluorescence-LIF spectroscopy, FBRM, and red green blue-RGB color imaging), process parameters, and raw material attributes are necessary to be included in the DF models [134,135]. Malechaux et al. demonstrated that a multiblock modeling approach was superior to hierarchical PLS-DA, as the simple concatenation of NIR and MIR data presented a small fraction of predictive variables compared to the complete dataset [11]. Other multiblock modeling methodologies are also promising, such as the response-oriented sequential alternation (ROSA), which facilitates handling many blocks [136]. It was also possible to include interactions in the model [137]. However, to the best of the authors’ knowledge, these approaches have not been utilized for real-life PAT problems.

For MLDF, it was demonstrated that both feature extraction and modeling steps significantly impact the model performance and, therefore, need to be optimized carefully [11,104]. For PAT data, a typical combination of methods is the application of individual PCA models for feature extraction and using the concatenated PC scores in a PLS regression model [72]. Besides PC scores, process/material parameters can also be conveniently incorporated into the PLS model, improving the model compared to the LLDF of the analytical sensor data [103]. Similarly, MSPC models can also be employed [10,103].

Another approach is the utilization of sequential methods in which the order of data blocks will be important for modeling. Most feature extraction procedures use an independent approach, meaning that each data source is processed individually, and the blocks are exchangeable. Foschi et al. used Sequential and Orthogonalized-Partial Least Squares-Discriminant Analysis (SO-PLS-DA) algorithm to classify samples through NIR and MIR data [138]. The algorithm builds a PLS model from the first data block and aims to improve the model’s performance using orthogonal (unique) information from the next data block. This sequential approach removes redundant information between datasets and extracts information to give an optimal model complexity [138].

After the features are derived from the raw data, ANNs can also serve as the DF model, which performed superior to PLS regression in multiple studies [104,105,139]. It was also possible to develop a cascade neural network using PCA scores to predict the quantitative process variables (i.e., component concentrations) of fermentation and then to evaluate the process state, e.g., determine the harvest time [140]. Compared to PLS, ANN and SVM have the advantage of being more suitable in the presence of non-linearity [85,104,141].

HLDF deduces a unique outcome from the results of multiple models, which are built with individual data sources. Consequently, the method requires decision support systems, which incorporate numerous versatile methods, e.g., sensitivity, uncertainty, and risk analysis [142]. Moreover, in the QbD concept, the design space is defined as the multi-dimensional combination and interaction of critical material and process parameters that are demonstrated to assure quality. That is, it could be regarded as an HLDF model when the critical input parameters are monitored with individual PAT tools and chemometric models. Design spaces could be defined by several methods, such as response surface fitting, linear and non-linear regression, first-principles modeling, or machine learning [143,144,145].

Independently of the fusion level, deep learning is another emerging modeling method for PAT data but has been neglected [62]. The structure of the deep neural networks enables the fusing of raw data (low-level), extracting features (mid-level), and making decisions (high-level) adaptively in a single model [146]. Several deep learning solutions can be found in the literature for DF in different industrial processes but not yet for pharmaceutical processes. For example, convolutional neural networks (CNN) could be used for fault diagnosis [147,148] or soft sensing in the production of polypropylene [149]. It has also been demonstrated that support vector machines, logistic regression, and CNNs could be used to fuse laser-induced breakdown spectroscopy (LIBS), visible/NIR hyperspectral imaging, and mid-IR spectroscopy data at different levels [119]. Therefore, their applications in pharmaceutical tasks could be further studied in the future.

## 4. Integrating DF into PAT

Considering the multivariate nature of pharmaceutical manufacturing, the implementation of DF in PAT is expected to be highly beneficial. The manufacturing of a product with a predefined quality profile is known to be dependent on the interplay between raw material attributes, formulation variables, and process parameters. Although the product development strategy strives to reach robustness, the uncontrolled variation and complex interaction between input factors can introduce variability in the performance of the drug product. Therefore, to mathematically describe and accurately predict the quality of a batch, the fingerprint of that particular run can be the best predictor. The fingerprint of a batch can be considered as a collection of data that comprises all the variables starting from the attributes of raw materials down to the timely evolution of process variables or CQAs. Such complex datasets, presenting diversely structured data from different sources, can be fully exploited only by implementing DF strategies.

Some good examples of complex quality attributes can be the tableting performance of granules and the dissolution profile of an API from prolonged-release tablets. To accurately predict the tableting performance of granules, it is important to have input data that can detect variations in granule particle size, particle size distribution, moisture content, crystallinity, and lubricant distribution. It is less likely that one PAT instrument will take account of all these factors, but combining machine vision (particle size; particle size distribution), NIR (moisture content; lubricant distribution), and Raman methods (crystallinity variation) stands as a promising solution. Similarly, for the accurate prediction of dissolution profiles, it is essential to keep track of API particle size variations, content, and particle size of the release controlling polymer, tablet crushing strength, lubricant distribution, and other factors depending on the particularities of the product [91,102,105,135,145].

The currently available pharmaceutical DF based applications are limited, suggesting its slow integration into this field. DF has been successfully applied for classification purposes, here including excipient qualification studies based on physical characteristics (XRPD and particle size distribution data) [150], the identification of counterfeit products [96,120], and the detection of product quality deviations [104].

The majority of process control applications dealt with the development of statistical process control methods (MSPC, BSPC) relying on continuously recorded univariate variables. Studies have been published on classical granulation [139,151], continuous granulation processes [90,92,93]; continuous tableting lines [131,152], and biotech processes [153,154,155]. On the other hand, studies that combine uni- and multivariate data are scarce. Bostijn et al. used MLDF to combine Raman spectroscopic data with univariate variables to monitor the manufacturing of an ointment type product and to reach an enhanced process control [156]. Probably, the challenges, with respect to the integration of multi- and univariate data into process control models, have limited the combined use of spectroscopic and classical process variables for the real-time monitoring of process evolution. Such an approach requires a specialized IT infrastructure for data collection, processing, and modeling. Thus, these elements have to be considered an integral part of a modern manufacturing line.

In the case of regression applications, the used modeling approaches reach a higher level of complexity when referring to the selected input variables. These studies usually predict CQAs of final/intermediate products or CPP setpoints for subsequent processing steps using a diverse range of input data. The first category of applications used the process fingerprint, represented by the timely evolution of univariate variables, to predict the desired responses [139,157,158]. The second category of applications used as predictors variables that do not evolve over time. To this respect, process conditions, raw material attributes, and multivariate data (spectroscopy) have been fused to predict granule quality [159], content uniformity [104,134], powder flowability [134], coating thickness [135], and the dissolution of the API [91,102,105,135,145].

The following parts of this section will focus on the key considerations regarding the development and validation of DF models, respectively, on their role within Pharma 4.0.

### 4.1. Model Development

The majority of the studies included in the literature survey have demonstrated the advantage of increased model performance by implementing DF, with less than 2% demonstrating similar results to individual models. As in most cases, adjustments made in the variable selection, feature extraction, type of the model, and DF strategy have led to considerable improvements in predictive performance; all these operations have to be thoroughly investigated during implementation.

A primary condition for reaching optimal model performance is to have relevant input variables. Thus, the decision to implement a DF strategy should start in the initial phases of the product’s lifecycle. Based on the results of risk assessment, the data collection strategy can be defined, deciding what data and which sensors are to be implemented on the manufacturing line. Moreover, an IT infrastructure has to be integrated into the process control strategy to efficiently handle incoming data from different sources/process steps. During product development, several data sources and PAT tools can be screened and ranked based on their usefulness in the model.

Before fusing data from multiple sources, it is essential to evaluate the contribution of each dataset to the model and its complementarity. Including this step into the model development routine can provide an estimation for the size of predictive data and uncorrelated variables, which can further justify the use of variable selection or feature extraction procedures. Ultimately, it can guide the correct choice of the best fusion strategy. The performance of fusion strategies with respect to the structure of input data and model objective was thoroughly described in Section 3.1.

Around 50% of the surveyed applications resumed the complementarity assessment to the comparison of various models built on individual data and fused datasets, here testing multiple strategies in a trial and error approach (Appendix A, Table A1). Studies that worked with univariate sensors did not evaluate this aspect, while others presented only one modeling approach. Approximately 20% of studies dedicate attention to the effective comparison of individual datasets. In this respect, methods such as statistical total correlation [160], correlation maps [128], pairwise correlation analysis [110], Pearson correlation analysis [114], confusion matrices [161], exploratory data analysis (EDA) [103], PCA [107,118,138], VIP [11], Hoteling’s T2 [104], MB-PLS—block importance evaluation [134,135,162], and OPLS [104] have been used.

As highlighted under Section 4, spectroscopic data represents a key input data source when considering pharmaceutical applications. The high throughput, non-destructive, and multivariate nature of these PAT tools are just some advantages that make them indispensable for reaching a more in-depth process control and product knowledge. Spectroscopic data, recorded over a few hundred wavelengths, is frequently used in the pharmaceutical field to predict CQAs and monitor production processes [163,164,165,166,167,168,169,170]. Fusing spectral data with other input variables will most likely require an MLDF approach, thus the identification of a suitable data processing and feature extraction procedure is key. To this respect, the application of PLS in DF has been extended towards developing models able to predict some key characteristics. In this manner, a large number of variables from spectroscopic data have been used to extract features such as moisture content, viscosity, acidic number [10], API concentrations in semisolid products [156], or the API and release rate controlling polymer content from prolonged-release tablets [145]. In subsequent steps, this meaningful process information (in the form of CQAs or performance parameters) has been used to detect deviations from normal process evolution or to predict batch quality. De Oliviera et al. highlighted the improved interpretability of such models compared to latent variables [10]. Other relevant outputs for spectroscopic data can be represented by concentration profiles estimated through MCR and Hoteling T2/Q residual-based indicators from MSPC models [10].

The model development step should be performed simultaneously/in parallel with the optimization of the data processing and complementarity testing, as these steps are highly interrelated. Further details on data processing and modeling opportunities were described in Section 3.2 and Section 3.3.

### 4.2. Model Validation

Implementing DF strategies for PAT purposes within the strict and highly regulated pharmaceutical environment will require extensive validation and robustness testing. Approximately 77% of the surveyed articles used an external dataset to test the developed models’ performance, while the remaining fraction relied on cross-validation procedures (Appendix A, Table A1). Testing the predictive ability of the models on external datasets is critical for performance evaluation purposes. Additionally, eight studies also evaluated the robustness of predictions by including controlled disturbances/interfering factors not considered in the calibration set (Appendix A Table A1**)**. Out of the surveyed articles, two studies particularly stand out regarding the validation procedure. First, Assis et al. evaluated the trueness, precision, linearity, and the working range of a NIR- and TXRF-based method used to assess the composition of roasted and ground coffee [77]. Second, Casian et al. used an accuracy profile approach to validate a four instrument DF platform used to predict the API content of electrospun nanofibers [104].

Although several DF applications are present in the literature, to the best of the authors’ knowledge, no studies have addressed the question of model validation and maintenance from the regulatory point of view, where different challenges arise depending on the fusion level. Nevertheless, the revised general chapter ‘Chemometric methods applied to analytical data’ (5.21) of the European Pharmacopoeia (Ph. Eur.), effective as of 1 April 2023, will include a new subsection dealing with DF [171]. This is expected to further promote the application of DF in the pharmaceutical industry.

The validation of an LLDF model is the most straightforward, as a single chemometric model is developed and validated. This is directly addressed, e.g., by the NIR guidance of the FDA or EMA [172,173]. Both guidelines consider NIR spectroscopy a suitable method for qualitative (identification/qualification) and quantitative analysis.

The papers integrate the terminologies and principles defined in ICHQ8-Q10. It is generally considered that NIR applications should use in the development strategy the principles of QbD based on risk assessment, conducted as per the ICHQ9 guideline, and both apparatus and material and manufacturing process-related variables should be considered. For risk control and mitigation throughout product lifecycle management, a DoE approach might be considered, and a risk assessment summary should be submitted to the regulatory authorities.

The validation requirements differ whether the NIR spectroscopic method is intended for qualitative or quantitative purposes. This could be transposed for fusion applications as well. Both guidelines have the minimum requirement of specificity and robustness for qualitative analysis.

The iterative nature of NIR method development should be kept-in-sight throughout product lifecycle management as new, other sources of variability can appear in future prediction sets. This implies a periodical re-evaluation of the method to confirm its suitability for the intended purpose and to be able to discriminate the out-of-specification (OOS) results. In the case of OOS results, a root cause investigation is necessary. If the outcome reveals that the OOS result is related to human or instrument error and the product complies using the reference method, the batch can be released. Until the update of the NIR method, its use should be suspended. From a regulatory perspective, minor modifications or those that are made within the scope of the elaborated NIR procedure should generally be handled by the pharmaceutical quality system of the Applicant under the principles of the current Good Manufacturing Practice (cGMP). Moderate, major, or modifications outside the scope of the elaborated NIR procedure implies the application of variation. A similar approach should be implemented for fusion-based analytical platforms.

These guidelines also emphasize that the selected variable range should be justified. For the LLDF model, this also means that the need for the DF should be confirmed, e.g., by comparing the performance of the data fused model to the models using a single PAT tool. Furthermore, the robustness testing and change control of the LLDF model might also impose a challenge, as the change or malfunction of a single PAT tool impacts the entire model. The utilization of sensitivity analysis or an MB-PLS model with block variance indices can assist these studies. It is also essential to establish a data quality management method for each PAT measurement (e.g., acceptance limits of similarity indices), as well as contingency plans for the potential failure of each PAT tool. This might be the usage of a chemometric model with the functioning analytical tool(s) if the analytical tools complement each other sufficiently.

A possible approach for validating an MLDF model might include the validation of multiple sub-models, i.e., the feature extraction models (e.g., PCA models), as well as the DF model. Consequently, if the acceptable ranges of inputs for the DF model are determined, the robustness of the individual models could be individually studied, and the change control procedure might be simplified, as in this case, it impacts only one sub-model. It is also worth noting that special attention must be paid during validation to justify the need for DF (similar to the low-level fusion) and the appropriate selection of features (e.g., number of PCs) to avoid the over-/under- fitting of the models.

As for the HLDF model, the validation and model maintenance of the individual models providing the input for the DF model is not affected by the DF. Hence the existing regulatory guidelines (e.g., the NIR guidance of FDA/EMA) could be directly followed. As mentioned in Section 3.3, the HLDF model could be regarded as a design space. Therefore, its construction, validation, and maintenance could be conducted following the ICH guidelines dealing with the QbD concept [174]. For example, the risk assessment steps, determining the acceptable ranges of the input parameters (CPPs and CQAs), and the edge of failure of the design space might be an integral part of the model validation.

### 4.3. DF and Pharma 4.0

The fourth industrial revolution in the pharmaceutical domain, known as Pharma 4.0, is set to streamline drug manufacturing through real-time optimization/control systems and fast decision making [175]. Pharma 4.0 will yield integrated, self-organizing, and autonomous manufacturing facilities, bringing the potential of more in-depth product- and process-related knowledge [176]. The smart factory will provide an improved control opportunity due to the greater connectivity and transparency by integrating digital solutions. The manual processes of classic manufacturing will be replaced by self-regulating automatic systems, improving the consistency in the quality of delivered products [177]. Reaching this level of technology requires the adoption of advanced data analytics and automation systems [175].

Data science was described as a core component of several Pharma 4.0 ideas. Although most data science tools are already available, they are not fully exploited, as they are applied to individual unit operations or subparts of the product lifecycle. The more powerful use of these tools for the materialization of autonomous systems stands in the development of interconnections between sensors and equipment from all unit operations [178]. The DF techniques combined with AI or machine learning can support the decision-making based on key performance indicators in industrial chemical plants [179]. Platforms are available, in which in silico development and optimization are performed by data-driven models and digital twins for pharmaceutical systems [180].

By working with different data sources and types, the data analysis procedure will be of key importance, especially since it will fuel the adaptive process control loops [176]. Thus, fusion-based data integration is needed to enable real-time monitoring and responsiveness within a well-controlled manufacturing environment. The collected data will gain digital maturity, as it is processed into actionable wisdom that can support decision-making [176,177].

The importance of data management solutions for data collection, organization, and integration is also acknowledged. Advanced computing infrastructure is needed to provide product or process-related information rapidly [175].

## 5. Future Perspectives and Final Remarks

The value of DF in the pharmaceutical industry was demonstrated in this work through the review of the existing literature in this field. The readily available large amounts of data coming from manufacturing processes are still not fully exploited to reach the status of a smart facility, as described in Pharma 4.0. In this respect, multiple obstacles need to be overcome.

Initial risk analysis during product development has to be extended to identify the most relevant data sources that can be used to gain a deeper level of knowledge and control for the developed product. Once the opportunity of implementing DF has been confirmed, the screening of instruments/sensors will be important to identify complementarity between different types of data. The use of complementary data sources will directly impact the performance of predictions and the efficiency of the implemented monitoring strategy.

On the technical side, efficient data management solutions have to be integrated into the manufacturing line to enable the real-time data processing needed for fast decision-making. Although the data processing and modeling tools are readily available, the improved connectivity between different unit operations still has to be resolved.

The slow integration of DF methods can be also explained, considering the strict regulatory environment of the pharmaceutical industry. On the other side, the already available and newly coming regulatory guidelines will strongly support pharmaceutical companies in this respect. Moreover, another major driving force is the fourth industrial revolution, Pharma 4.0, where DF occupies a key position for an efficient implementation.

## Appendix A

**Table A1:** Literature survey on the use of data fusion for classification, process control, and regression applications.

Domain	Objective	Data Source	Data Fusion Level	Modeling Method	Variable Selection/Feature Extraction	Complementarity Evaluation	Performance Results	Robustness/Validation	Reference
**CLASSIFICATION**									
Agriculture	Discrimination of different crop types	CCD digital camera; Spectro-radiometry	MLDF	DISCRIM (SAS)	PCA	/	MLDF > individual model	/	[181]
Botanical	Plant recognition	Spectro-radiometer; Imaging	MLDF	Euclidean distance	Spectral signatures; leaf venation feature extraction	DF-individual model comparison	MLDF > individual model	e.d.	[182]
Chemical	iIdentification of essential oils in Melaleuca sp.	GC-MS; NMR	LLDF	-	-	Statistical Total Correlation	LLDF > individual model	/	[160]
	Classification of pigments and inks	LIBS; Raman	LLDF	PCA; SIMCA; PLS-DA; SVM	-	DF-individual model comparison	LLDF > individual model	/	[183]
	Identification of explosives	Raman; LIBS	MLDF	Simple Linear correlation	2D-image based estimator	DF-individual model comparison	MLDF > individual model	/	[95]
	Classification of ochre pigments	Micro-Raman; XRF	LLDF; MLDF	PLS-DA	PLS-DA-based identification of the local positive maxima and negative minima of the weights for variables with good classification power	DF-individual model comparison	MLDF > LLDF	e.d.	[80]
Environmental	Evaluate the state of conversion over time for an ecosystem	Conductivity; pH; NIR; Fluorescence emission-excitation data	MLDF	MCR-ALS	PARAFAC; MCR-ALS	/	MLDF > individual model	/	[184]
	Quantify potentially toxic elements from soil	NIR; TXRF	LLDF; MLDF	SVM	UF; GA	DF-individual model comparison	response dependent; GA > UV	e.d.	[113]
Food	Chestnut cultivar identification	Sensory evaluation; FT-NIR	LLDF	PLS-DA	-	DF-individual model comparison	LLDF > individual model (response dependent)	/	[185]
	Authentication of raw and cooked free-dried rainbow trout fillets	NIR; Colorimetry; Texture analysis	LLDF	PLS-DA; LDA; QDA; kNN	-	DF-individual model comparison	LLDF > individual model	e.d.	[186]
	Classification of sparkling wines	HPLC; antioxidant capacity tests; FTIR	LLDF	PCA; HCA; PLS-DA	-	/	LLDF > individual model	e.d.	[187]
	Detect adulteration of cocoa butter	Fluorescence; UV	LLDF	PCA-LDA	-	DF-individual model comparison	LLDF > individual model	e.d.	[188]
	Storage time classification	Dielectric spectroscopy; Computer Vision	MLDF	ANN; SVM; BN; MLR	CFS; image processing—red, green, blue, hue, saturation, intensity, lightness, a∗ and b∗ chromatic components	/	MLDF > individual model	e.d.	[189]
	Understand the effect of storage factors on rice germ shelf life	NIR; e-nose	MLDF	PCA	PLS (NIR); Pearson’s correlation coefficient-based data selection (e-nose)	Correlation maps	no comparison	/	[128]
	Characterisation of black pepper	LC-MS; GC–MS; NMR	MLDF	OPLS-DA	OPLS-DA -> VIP	DF-individual model comparison	enhanced process control	e.d.	[111]
	Discrimination of four species of Boletaceae mushrooms from different geographical origins	UV-VIS; FT-IR	MLDF	PLS-DA; GS-SVM	PLS-DA	DF-individual model comparison	MLDF > individual model; GS-SVM > PLS-DA;	e.d.	[190]
	Predict fish freshness through total volatile basic nitrogen level	NIR; Computer Vision	MLDF	BP-ANN	PCA	DF-individual model comparison	MLDF > individual model	e.d.	[126]
	Predict the olive variety	E-nose; E-eye; E-tongue	MLDF	PLS-DA	PCA	DF-individual model comparison	MLDF > individual model	e.d.	[191]
	Classification of edible salts	DORS; LIBS	MLDF	PCA; kNN	PCA	confusion matrices	MLDF > individual model	e.d.	[161]
	Authentication of virgin olive oil	CE-UV; GC-IMS	HLDF	PCA, LDA, kNN	-	DF-individual model comparison	HLDF > individual model	e.d.	[192]
	Craft beer authentication	Thermo-gravimetry; MIR; NIR; UV; VIS	LLDF; MLDF	SIMCA; PLS-DA	PLS-DA scores on individual data sets	sensitivity & specificity comparison	MLDF > LLDF	e.d.	[74]
	Establish the geographical traceability of wild Boletus tomentipes	FT-MIR; ICP-AES data recorded on two parts of the mushroom (pileus and stipe)	LLDF; MLDF	SVM; RF	PCA	DF-individual model comparison	MLDF > LLDF	e.d.	[83]
	Discrimination of emmer landraces	NIR; MIR	LLDF; MLDF	PLS-DA; SO-PLS-DA	Scores of optimal single-block PLS-DA or multiblock	PCA	MLDF > LLDF	e.d.	[138]
	Varietal discrimination of olive oil	NIR; MIR	LLDF; MLDF; HLDF	PLS-DA; Decision HLDF:majority vote	PLS-DA; MB-PLS-DA	VIP- evaluate variable contribution	HLDF > individual model; MLDF >/< individual model (f. methodology); LLDF ≈ individual model;	e.d.	[11]
	Identification of the botanical origin of honey	IR; NIR; Raman; PTR-MS; E-nose	LLDF; MLDF; HLDF	PLS-DA (Decision HLDF: indiv PLS-DA—majority voting and Bayesian consensus with discrete probability distributions)	PCA	DF-individual model comparison	HLDH > MLDF/LLDF	e.d.	[121]
	Authentication of Panax notoginseng geographical origin	FT-MIR; NIR	LLDF; MLDF; HLDF	RF	RF; PCA	DF-individual model comparison	HLDF > MLDF > LLDF	e.d.	[82]
	Detect the adulteration of hazelnut paste with almond	NIR; Raman	MLDF; HLDF	SIMCA	variable selection based on the normalized differences between reference and sample spectral data	DF-individual model comparison	MLDF > HLDF	e.d. + interfering factors	[89]
Medical	Diagnosis of lung cancer	FT-IR; Raman	LLDF	PLS-DA	-	DF-individual model comparison	LLDF > individual model; Wavelet threshold denoising of spectral data was beneficial	e.d.	[193]
	Discrimination of raw and processed Curcumae rhizoma	FT-NIR; E-nose; colorimetry	MLDF	PLS-DA	GA -> PLS; IRIV -> PLS; CARS -> PLS for NIR; correlation coefficient based feature extraction for e-nose	Pairwise correlation analysis	MLDF > individual model	e.d.	[110]
	Identification of rhubarb	NIR; MIR	MLDF	PLS-DA; SIMCA; SVM; ANN	Wavelet compression; iPLS	PCA	MLDF > individual model	e.d.	[107]
Pharmaceutical	Evaluate nanofiber deposition homogeneity	NIR; Raman; Colorimetry; Image analysis	MLDF	PLS; ANN	PCA/OPLS scores from raw or preprocessed data	Hoteling’s T2	MLDF > individual model	e.d.	[104]
	Identification of counterfeit pharmaceutical packaging	LIBS; ATR-FTIR	MLDF	kNN; LDA	PCA	DF-individual model comparison	MLDF > individual model	e.d.	[96]
	Omeprazole fingerprinting to detect counterfeit products	HPLC-UV; GC-MS; NIR; NMR; XRPD	LLDF; MLDF	PCA; HCA	PCA	DF-individual model comparison	DF > individual model (f. fused data)	/	[120]
	Advanced qualification of pharmaceutical excipients	XRPD; PSD data	LLDF; MLDF	MB-PLS	-	Predict LV’s of one method using data originating from other sources	DF > individual model	e.d.	[150]
**PROCESS CONTROL**									
Automotive	Establish good and stable operating conditions for an autobody assembly process	Seal gap; margin and flushness measurements	LLDF	PCA	-	/; complementary univariate sources	Enhanced process control	/	[194]
Chemical	Control polymer properties	Temperature sensors; feed rate	LLDF	PLS	-	/; complementary univariate sources	Enhanced process control	/	[195]
	Monitor the conversion of nitrobenzene to aniline	UV spectroscopy; process variables (reactor temperature, reactor pressure, gas feed, jacket in/out temperature, oil flow rate, stirrer speed)	LLDF	PLS	-	DF-individual model comparison	Enhanced process control	e.d.	[196]
	Process management and end-point identification	Temperature; Pressure; Flow rate	LLDF	MPLS	-	/; complementary univariate sources	Enhanced process control	/	[197]
	On-line monitoring of injection molding and a fed-batch penicillin cultivation process	7 univariate process variables for injection molding; 10 univariate process variables for cultivation process	LLDF	PCA; DPCA; MPCA	-	/; complementary univariate sources	Enhanced process control	e.d. + interfering factors	[198]
	Multivariate monitoring of a continuous API synthesis	29 process variables for stage 1; 40 process variables for stages 2–3	LLDF	PCA	-	/; complementary univariate sources	Enhanced process control	e.d.	[199]
	Fault detection for a sulfite pulp digester process	Temperature; pressure; viscosity; Kappa number	LLDF	PCA	-	/; complementary univariate sources	Enhanced process control	/	[181]
	Monitoring of a polymer reactor in a petrochemical plant	Not presented	LLDF	PCA	-	/	Enhanced process control	e.d.	[200]
	Monitoring of tryptophan and biomass for bioprocess production	E-nose; NIR; standard bioreactor probes	MLDF	PLS	Forward selection procedure based variable selection relying on the correlation with the desired model output	/	Enhanced process control	e.d.	[201]
	Monitor the solid-state fermentation process of feed protein; process state identification	E-nose; NIR	MLDF	BP-AdaBoost neural network	PCA; ICA	DF-individual model comparison; PCA	Enhanced process control	e.d.	[118]
Food	Analyze the continuous bottling process of beverages	CO2 content; sugar content; Net content; washer temperatures; rinse temperatures; closing/opening torque	LLDF	PCA; 3-way PLS	-	/; complementary univariate sources	Enhanced process control	/	[202]
Pharmaceutical	Multivariate monitoring of continuous tableting line	37 process sensors from 5 unit operations	LLDF	PCA; PLS	-	/; complementary univariate sources	Enhanced process control	e.d.	[131]
	BSPC of a continuous twin-screw granulation line	21 process parameters related to multiple unit operations	LLDF	PLS	-	/; complementary univariate sources	Enhanced process control	e.d.	[92]
	MSPC of a continuous granulation and drying process	25 univariate variables logged by ConsiGmaTM	LLDF	PCA	-	/; complementary univariate sources	Enhanced process control	e.d. + interfering factors	[93]
	MSPC of a continuous granulation and drying process	35 univariate variables for the monitoring of granulation and drying	LLDF	PLS	-	/; complementary univariate sources	Enhanced process control	e.d.	[90]
	Multivariate control of continuous tableting line	14 univariate variables recorded from feeding, extrusion, and drying unit operations	LLDF	PCA; PLS	-	/; complementary univariate sources	Enhanced process control	/	[152]
	MSPC of a granulation process	temperature; agitation speed; torque; power consumption	LLDF	PLS	-	/; complementary univariate sources	Enhanced process control	/	[151]
	Predict culture performance across different scales	pH; dissolved oxygen; temperature; dissolved CO_2_; metabolic indicators; cell growth parameters	LLDF	PLS	-	/; complementary univariate sources	Enhanced process control	/	[153]
	Monitoring of a fed-batch cell culture process	pH; agitation; air/CO_2_/O_2_ flows; dissolved O_2_; vessel temperature	LLDF	PCA	-	/; complementary univariate sources	Enhanced process control	e.d. + interfering factors	[154]
	Batch modeling of cell culture unit operation	pCO_2_; pO_2_; glucose; pH; lactate; ammonium ions	LLDF	PLS	-	/; complementary univariate sources	Enhanced process control	/	[155]
	Process control for ointment manufacturing	Temperature; Viscosity; FBRM; Raman (API concentration)	MLDF	PLS	PLS	/	Enhanced process control	e.d. + interfering factors	[156]
Pharmaceutical/Chemical	MSPC of fluid bed granulation, polyester production, and gasoline distillation processes	Temperature sensors; NIR;	MLDF	PCA	PLS; MCR-ALS; T2, Q—derived from NIR based MSPC	/	Enhanced process control	e.d.	[10]
**REGRESSION; PROCESS CONTROL**									
Chemical	Monitor glucose concentrations on a fermentation process	NIR; airflow rate; alkali addition rate	LLDF	PLS	SWS	DF-individual model comparison	LLDF > individual model	/	[203]
	MSPC control chart for styrenic polymer production process; predict melt flow index and percentage of bound acetonitrile	NIR/NIR; process sensor data*	LLDF; MLDF*	PLS; PCA	PCA	EDA	MLDF > individual model	e.d.	[103]
Food	Monitoring of yogurt fermentation	NIR; temperature; E-nose	MLDF	ANN	Forward selection	/	No comparison	e.d. + interfering factors	[140]
Pharmaceutical	BSPC of a fluid bed granulation process; prediction of granule density and flowability from process fingerprint	Spatial filter velocimetry; temperature data	LLDF	PLS	-	/	Enhanced process control	e.d.	[157]
	Predict granulation water, tableting speed, and tablet disintegration	Process data; Raw material data; Granulometric data	MLDF	PLS; ANN	PCA	/; complementary univariate sources	Enhanced feedforward process control	e.d.	[139]
	Predict the viscosity of a personal care product from process data	8 process parameters (temperature, pressure data)	MLDF	MPLS	PLS	/; complementary univariate sources	Enhanced process control	/	[158]
**REGRESSION**									
Agriculture	Determination of starch and protein content in navy bean flour	NIR; Fluorescence spectroscopy	LLDF	PCR; PLS	-	X-Y correlated variability estimated	LLDF >/≈ individual model (f. response)	e.d.	[204]
Chemical	Analysis of protein secondary structure	CF; UVRR	LLDF	MCR-ALS	-	DF-individual model comparison	LLDF > individual model	/	[205]
	Analysis of coal volatile content and caloric value	LIBS; FT-IR	LLDF	PLS	-	DF-individual model comparison	LLDF > individual model	e.d.	[206]
	Simultanous determination of Cu(II), Ni (II) and Cr (II)	UV-VIS spectroscopy	MLDF	PLS	Wavelet transformation	Fusion of different scale based wavelet coefficients	MLDF > individual model	e.d. + interfering factors	[115]
	Prediction of elemental concentrations in ore	MWIR; LWIR	LLDF; MLDF	PLS	PCA	DF-individual model comparison	LLDF > individual model > MLDF	e.d.	[72]
	Determination of deltamethrin in insecticide formulations	NIR; UV-VIS	LLDF; MLDF	ELM	PLS	DF-individual model comparison	LLDF > MLDF/individual model	e.d.	[76]
	Predict properties of oil/biodiesel blends	NIR; MIR	LLDF; MLDF	PLS; SVM	VIP -> PCA; iPLS -> PCA	DF-individual model comparison	DF > individual model	e.d.	[100]
Environmental	Predict total carbon and nitrogen in soil samples	PXRF; VIS-NIR	LLDF	RF; PSR	-	DF-individual model comparison	LLDF > individual model	e.d.	[207]
	On-line mineral identification of tailing slurries of an iron ore concentrator	LIBS; NIR; XRF	LLDF	PLS	-	DF-individual model comparison	LLDF > individual model	/	[208]
	Predict soil texture	PXRF; NIR	MLDF	PLSR; SMLR	PCA	DF-individual model comparison	MLDF > individual model	e.d.	[127]
Food	Age time prediction of wine	FT-IR; UV-VIS; Colorimetry	LLDF	PLS	-	DF-individual model comparison	LLDF ≈ individual model	e.d.	[209]
	Determination of micro and macroelements in Brachiaria forages vegetal samples	NIR; LIBS	LLDF	PLS	rPLS	DF-individual model comparison	LLDF > individual model	e.d.	[106]
	Predict the K, Mg, and P concentration in bean seeds	LIBS; WDXRF	LLDF	MLR	-	DF-individual model comparison	LLDF > individual model	e.d. + interfering factors	[210]
	Characterization of crude oil products	IR; Raman; NMR	LLDF	PLS	-	DF-individual model comparison	LLDF > individual model	e.d.	[98]
	Prediction of quality indices	Dielectric spectroscopy; Computer Vision	MLDF	ANN; SVM; BN; MLR	CFS; image processing—red, green, blue, hue, saturation, intensity, lightness, a∗ and b∗ chromatic components	/	MLDF > individual model	e.d.	[189]
	Predict the yield of drought stressed spring barley	NIR; thermal and distance measurements	MLDF	PLS; MLR	Calculation of spectral indices	/	MLDF > individual model	/	[211]
	Predict the freshness of pork meat	Spectral and textural data extracted from Hyperspectral images	MLDF	PLS	Spectral waveband extraction; SPA; texture extraction—GLCM	DF-individual model comparison	MLDF > individual model	e.d.	[97]
	Predict the water holding capacity of chicken breast fillets	Spectral and textural data extracted from Hyperspectral images	MLDF	PLS	RC based wavelength selection; GLCM—texture variables;	DF-individual model comparison	MLDF > individual model	e.d.	[212]
	Predict the total volatile basic nitrogen level in fish fillet	Spectral and textural data extracted from Hyperspectral images	MLDF	PLS; LS-SVM	PN-GA	DF-individual model comparison	MLDF > individual model	e.d.	[141]
	Predict tenderness of porcine muscle	NIR; Computer Vision	MLDF	PLS	Discrete wavelength transformation (computer vision)	DF-individual model comparison	MLDF > individual model	e.d.	[213]
	Predict pH for salted meat	Spectral and textural data extracted from Hyperspectral images	MLDF	PLS	PCA (spectral data); GLCM (textural features)	DF-individual model comparison	MLDF ≈ individual model	e.d.	[214]
	Qualitative identification and quantitative prediction (amino acids, caffeine, polyphenols, catechins) of tea quality	E-nose; E-eye; E-tongue	MLDF	PLS; SVM; RF	PCA	DF-individual model comparison	MLDF > individual model	e.d.	[215]
	Quantitative evaluation of pesiticide residue in tea	Confocal Raman microspectroscopy; E-nose	MLDF*	PLS; SVM; ANN	VIP; iPLS; rPLS; GA; CARS; SPA	DF-individual model comparison	MLDF > individual model; ANN> PLS/SVM	e.d.	[216]
	Quantify the composition of roasted and ground coffee	NIR; TXRF	LLDF; MLDF	PLS	SVPII -> PLS; GA -> PLS; OPS -> PLS	DF-individual model comparison	LLDF > MLDF; SVPII > GA/OPS	e.d. + trueness, precision, linearity, working range	[77]
	Predict total volatile basic nitrogen content in chicken meat	Colorimetric sensor; optical sensor	LLDF; MLDF	PCA-BPANN	ILA; LLA-(hyperspectral data); Pearson’s correlation coefficient based variable selection;	Pearson correlation analysis	MLDF > LLDF; removing uncorrelated data improved results	e.d.	[114]
	Olive leaf analysis and crop nutritional status	FT-NIR; EDXRF	LLDF; MLDF	PLS	PCA	DF-individual model comparison; X-Y correlated variability estimated	MLDF > individual model; LLDF >/< individual model;	e.d.	[124]
	Moisture content prediction in the processing of green tea	Computer vision; NIR	LLDF; MLDF	PLS; SVR	RFg; CARS; VCPA-IRIV; color and texture features for images/CV	DF-individual model comparison	MLDF > LLDF	e.d.	[81]
	Predict the composition of coffee blends	ATR-FTIR; PS-MS	LLDF; MLDF	PLS	GA-> PCA; OPS -> PCA	DF-individual model comparison	LLDF > MLDF; OPS > GA;	e.d.	[78]
	Prediction of olive oil sensory descriptors	FT-MIR; UV-VIS; HS-MS	LLDF; MLDF	PLS	PLS	DF-individual model comparison	DF > individual model (f. response)	e.d.	[125]
	Quantification of Ca in infant formula	FT-IR; Raman	LLDF; MLDF	PLS	VIP -> PLS	DF-individual model comparison; individual data characterisation	MLDF > LLDF	e.d.	[84]
	Quantitative estimation of 10-hydroxy-2-decenoic acid in royal jelly samples	ATR-FTMIR; NIR	LLDF; MLDF	PLS	SI-PLS -> PCA; SI-PLS -> ICA	DF-individual model comparison	MLDF > LLDF	e.d.	[75]
	Predict the total antioxidant activity and total phenolic content of Chinese rice wine	ATR-IR; Raman	LLDF; MLDF	PLS; SVM	SiPLS -> PCA	DF-individual model comparison	MLDF > individual model > LLDF (more redundant info)	e.d.	[85]
	Predict the sensory attributes of rice wine samples	E-nose; E-eye; E-tongue	LLDF; MLDF	MLR; BP-ANN; SVM	PCA; MLR (crossperception DF)	DF-individual model comparison	Cross-perception DF > LLDF/MLDF/individual models	e.d.	[217]
	Age time prediction of wine	SFE-GC-MS; HPLC-DAD; LC-DAD; UV-VIS	LLDF; MLDF*	Concatenated PLS; MB-PLS; HPLS; NI-SL; SO-PLS	-	Block importance evaluation	multiblock DF > single block LV methods	e.d.	[162]
	Quantitation of rapeseed oil as contaminant in adulterated olive oil	NIR; MIR	LLDF; MLDF; HLDF	PLS/bi-linear regression for HLDF	SPA	DF-individual model comparison	HLDF > LLDF > Individual model > MLDF	e.d.	[79]
Pharmaceutical	Predict quality model for HSWG process-based formulations	Literature data; Process data in HSWG	LLDF	PLS	-	/; complementary univariate sources	LLDF > individual model	e.d.	[159]
	Predict Beta-carotene, Riboflavin, ferrous fumarate, ginseng, and ascorbic acid content in powder blends; quantify powder flow behavior	Light-induced fluorescence spectroscopy; NIR; RGB color imaging	LLDF	MB-PLS	-	MB-PLS	LLDF > individual model	e.d.	[134]
	Predict the thickness of microsphere coating and API dissolution performance	Raw material data; Process data; NIR; Raman; FBRM	MLDF	MB-PLS	-	MB-PLS	MLDF ≈ Raman individual model*	/	[135]
	Predict meloxicam content in nanofibers	NIR; Raman; Colorimetry; Image analysis	MLDF	PLS; ANN	PCA/OPLS scores from raw or preprocessed data	OPLS	MLDF > individual model	Accuracy profiles	[104]
	Dissolution prediction	Univariate Process Parameters; NIR	MLDF	PLS	PCA	DF-individual model comparison	MLDF > individual model	/	[91]
	Predict dissolution profile for sustained release tablets	NIR; compression force; PSD data	MLDF	ANN; SVM; ERT	PLS (tablet composition prediction)	DF models comparison	MLDF > individual model; ANN> SVM/ERT	e.d.	[145]
	Predict dissolution profile for modified release tablets	Reflection and transmission NIR; reflection and transmission Raman	MLDF	ANN; PLS	PCA	DF-individual model comparison	MLDF > individual model; ANN > PLS	e.d.	[105]
	Predict dissolution profile for immediate release tablets	NIR; formulation-material-process variables	MLDF	PLS	PCA	DF-individual model comparison	MLDF > individual model (f. response)	e.d.	[102]
